# From One to Many and Back Again: A Systemic Signal Triggers Tunicate Regeneration

**DOI:** 10.1371/journal.pbio.0050098

**Published:** 2007-03-06

**Authors:** Liza Gross

For a lucky subset of vertebrates, losing an appendage is no big deal. As many an inquisitive child knows, salamanders can regenerate lost limbs or tails; and as lab investigators know, zebrafish can regrow lost fins. Of course, humans and other “higher” vertebrates must make do with repairing rather than regenerating damaged tissues. Though whole body generation (WBR) does occur, it’s typically restricted to a subset of morphologically less complex invertebrates, such as sponges, flatworms, and jellyfish.

In a new study, Yuval Rinkevich et al. shed light on the molecular signals underlying WBR by investigating the phenomenon in our closest invertebrate relative, the sea squirt *Botrylloides leachi*. The researchers identified several novel features of WBR in this colonial marine organism, and discovered an unusual mode of regeneration.

As invertebrate members of the phylum Chordata, sea squirts share several fundamental biological pathways with vertebrates; consequently, using them as a model system to study WBR could illuminate not only the evolutionary origin of regeneration, but also its subsequent attenuation in vertebrates.

Sea squirts (also called “tunicates” after their tough outer tunic) are widely distributed in shallow coastal waters as colonies of genetically identical individuals called “zooids.” These clones develop from sexually reproduced larvae that adhere to rocks or other substrates on the sea floor soon after hatching. Once attached, the tadpole-like larvae lose several of the features that make them chordates—the notochord, dorsal nerve tube, and postanal tail (muscle tissue beyond the digestive tract)—and develop adult organs, including a digestive tract equipped with pharyngeal slits, endostyle, neural complex, heart, and gonads.

Thus transformed, the sedentary tunicate initiates the rounds of asexual reproduction that create the colony. During this four-stage process, called blastogenesis (or palleal budding), new buds sprout from the thoracic body wall of the founding “oozooid,” spawning zooids that repeat the process. As new buds mature, parental zooids degrade and undergo resorption into the colony. Zooids connect to colony mates through a network of blood vessels with delicate fingerlike projections called ampullae. Experiments in a close relative of *B. leachi* showed that buds forming at the base of these vascular ampullae supported WBR.

**Figure:**
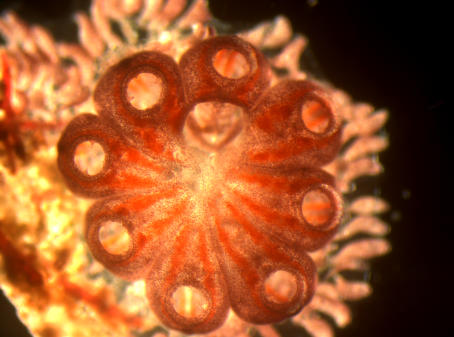
Recognized as the one of the closest relatives to vertebrates, the colonial urochordate Botrylloides leachi possesses unique modes for propagating and regenerating that make this species a robust model organism for studying diverse biological issues related to development, immunology, stem cells, and regeneration.

To investigate WBR in *B. leachi*, Rinkevich et al. collected colonies from the Mediterranean coast of Israel and analyzed the morphological, cellular, and molecular characteristics of the process. The researchers removed fragments of blood vessels with ampullae from the colonies, and placed the fragments on slides for regeneration. Of 95 fragments, 80 underwent WBR.

Following dissection, vessels within fragments contracted and blood flow abated; within a day, a new circulatory system emerged as vascular connections between ampullae grew and blood flow returned. Ampullae dynamically changed shape and position over the course of a week, creating a dense localized network with new blood vessels opposite a vessel-free gelatinous tunic matrix. Blood flow attenuated, and an opaque mass of vessels formed around a transparent vesicle with two openings. Within 10 to 14 days, this opaque mass yielded functional siphons (an inhalant, or peribranchial, siphon and an exhalant, or atrial, siphon) and a “fully operating filter-feeding zooid.” Among several regeneration starts, only the fastest developing bud in a fragment reached the final zooid stage; the others were absorbed into the colony.

Each day, Rinkevich et al. placed regenerating fragments under the microscope to study their cellular changes. By the second day, dozens of small compartments—the newly dubbed regeneration niches—started forming and filling with clusters of blood cells. Over the next few days, aggregating cells formed around a hollow sphere, then reorganized into a thin and thick layer on opposite sides, very similar to early stages of embryonic development. As cells proliferated, buds grew, and the thick cell layer folded inward, forming double-walled folds (which became the pharyngeal slits) and creating a middle chamber and two side chambers (which became the pharyngeal chamber and lateral atrial chambers). Organ development continued, and an adult zooid, capable of sexual reproduction, appeared within two weeks.

For molecular insights into regeneration, the researchers focused on retinoic acid (RA) signaling by examining the temporal expression of its receptor (RAR). In addition to its role in chordate body patterning, RA (a vitamin A metabolite) induces the regeneration of several tissues and organs. Only regenerating vessels and ampullae expressed RAR, and this expression continued through each phase of regeneration.

The researchers confirmed RA’s vital role in regeneration by inhibiting RA synthesis with chemicals and destroying RA transcripts with RNA interference. In both cases, malformed buds failed to generate zooids from dissected fragments. Similar problems occurred when RAR function was disrupted. In contrast, RA overexpression led to accelerated regeneration, with multiple buds reaching the fully developed zooid stage. RAR regulates developmental elements of the normal budding process in a sister colonial tunicate species, suggesting that organisms recruit the same signals for development and regeneration.

In regenerating fully functional adult tunicates from “minute vascular fragments,” the researchers identified several features of this system that differ from those of established regeneration model systems. In contrast to limb or fin regeneration—which arises from local signals emanating from a “regeneration center”—*B. leachi* WBR arises from systemically induced signals in multiple “regeneration niches.” These niches arise from the vascular network (rather than from proliferating balls of cells), and regeneration appears to be regulated by systemic (rather than local) cues. These systemic cues, the researchers propose, may travel through the circulation, thereby supporting multiple regeneration foci. The researchers plan to investigate the cellular source of the tunicate’s remarkable regenerative power in future studies.

